# Innovative analytical model for temperature prediction of front-end accessory drive

**DOI:** 10.1038/s41598-020-79986-5

**Published:** 2021-01-14

**Authors:** Xingchen Liu, Kamran Behdinan

**Affiliations:** Advanced Research Lab for Multifunctional Lightweight Structures (ARL-MLS), Department of Mechanical and Industrial Engineering, University of Toronto, Toronto, Canada

**Keywords:** Mechanical engineering, Pure mathematics

## Abstract

The front-end accessory drive belt drive system is a critical component in the vehicle engine. To avoid thermal deterioration under static state operating conditions, the thermal distribution for the belt drive system at each condition must be determined in an efficient manner. Due to the numerical approach is not feasible to address this concern because of its high computational cost, this paper proposes a reliable and efficient novel analytical thermal model to achieve this goal. This work develops the state-of-the-art heat transfer ordinary differential equations (ODEs) describing the thermal flow and heat dissipations on the complex structures of pulleys. Then it integrates these ODEs with heat transfer governing equations of the belt and heat exchanges to establish an innovative system of equations that can be solved within a few seconds to provide temperature plots. Moreover, experiments were conducted on a dynamometer to verify the accuracy of the proposed model under a wide range of conditions. The results indicate that the measured temperatures are in good agreement with the corresponding analytical results. Owing to its efficiency, the proposed model can be integrated with other mechanical characterizations of the belt drive system in terms of design, optimization, and thermal fatigue analyses.

## Introduction

**Figure 1:**
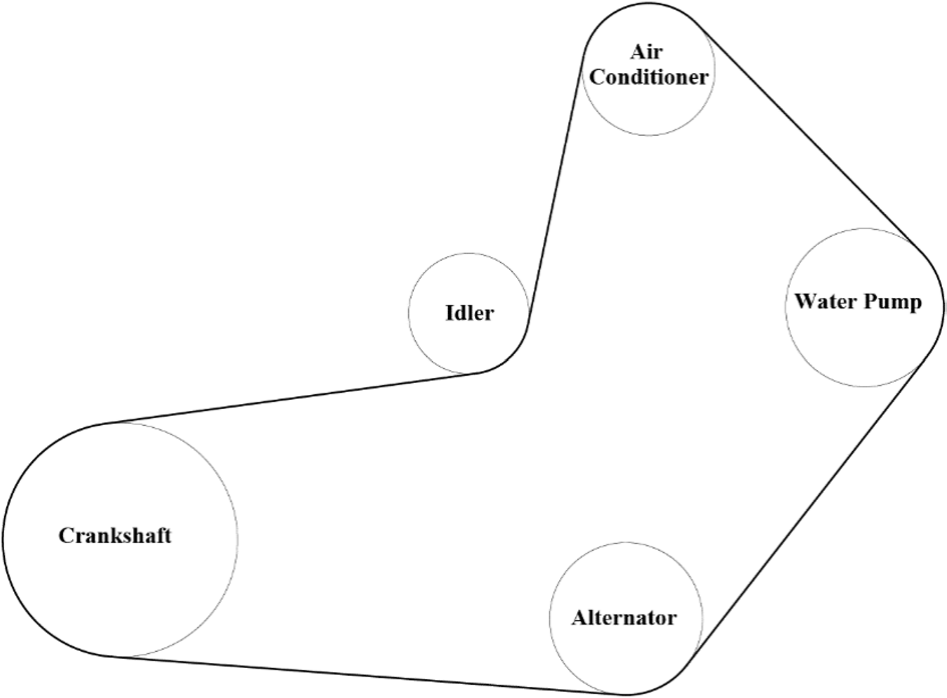
Car engine and FEAD system^3^.

A front-end accessory drive (FEAD), as shown in Fig. 1, is a critical belt drive system in an automotive engine. It consists of a driving pulley (DR pulley), a belt, and a number of driven pulleys (DN pulleys). An engine crankshaft drives the DR pulley, inputs energy into the belt drive system, and transmits energy through the belt to DN pulleys. Then, these pulleys drive other parts of the engine, such as a water pump or an alternator, to maintain engine operation. Currently, the DN pulley material, which is a kind of fiber-reinforced plastic (FRP) with low thermal conductivity^1^, is prone to deterioration and blistering under high temperature in the under-the-hood environment and a large amount of heat generation within the FEAD system during engine operation. Eventually, this deterioration causes pulley structural failure. To overcome this problem, this study developed a thermal analytical model that can predict real-time temperatures for target locations on the belt and pulleys to prevent material thermal failure under engine operating conditions. Unlike computationally expensive methods such as computational fluid dynamics^2^, this model functions as a fast and economical tool to provide black-box analysis for subsequent research investigations. For example, this analytical model can rapidly provide temperature variations under numerous operating conditions, enabling material scientists to address the thermal fatigue of FRP and thus improve its reliability.

The first step of thermal analysis for a belt drive system is to confirm the heat generation flux and its locations. A number of studies related to the power loss of the belt drive system have focused on improving the efficiency of the belt drive system. However, the power loss can also provide the heat generation within the system in this case. One prevalent theory for V-rib rubber belt drive systems considers five forms of energy loss based on V-belt movement mechanisms, namely, belt bending, stretching, shear, radial compression, and slip^4^. Manin et al.^5^ extended this method to a multi-pulley serpentine belt transmission system. Silva et al.^6,7^ considered that primary power losses are caused by belt hysteresis, which can be classified as the bending, stretching, shear, flank, and radial compression of belt rubber, and applied this theory to a poly-V belt drive. Bertini et al.^8^ developed an analytical power loss model by considering belt sliding, hysteresis, and engagement/disengagement frictional losses. Chen et al.^9^ experimentally investigated the power loss of a rubber V-belt based on various speed ratios, belt tensions, rotational speeds, external loads, and diameters of a pulley. Various factors may influence the belt drive mechanism and its power loss; one such factor is belt slip. Balta et al*.*^10^ used the response surface method to establish a relationship between belt slip behavior and belt drive parameters under various operating conditions. The transverse dynamic hysteretic damping characteristics of a serpentine belt were estimated by utilizing the variable stiffness and damping model^11^. Kim et al.^12^ developed a belt force equation based on the classical Euler formula to predict the normal and tangential belt force distribution of a flat belt drive. Qin et al.^13^ developed an analytical model for the friction damping of round clamp band joints, which can predict the energy dissipation caused by the friction contact between joint components. Shen et al.^14^ used a dynamic finite element model (FEM) to simulate belt contact deformations and belt–pulley interaction, which can potentially be used to predict transient power loss. Other studies have focused on the power loss of another type of belt drive system, i.e., continuously variable transmission (CVT). Julió et al.^15^ presented a model to predict the transmission ratio time response for the rubber belt CVT under various operating conditions. This model can be used for CVT design and optimization. Zhu et al.^16^ studied the torque and speed loss of the rubber V-belt CVT based on its mechanism. Several methods have been developed to improve the efficiency of this type of CVT.

The second step of thermal analysis is to use the information of power loss or heat generation to perform heat transfer analysis. Wurm et al.^17,18^ simulated the heat transfer analysis for a CVT system using CFD under critical load cases. They used a novel method to simulate rotational symmetric temperature profiles for nonrotating pulleys in the numerical program. Gerbert^19^ proposed a static analytical thermal model for a simple flat two-pulley-one-belt drive system. This model established a set of ODE equations for the heat conduction within the components and the heat exchange of belt–pulley contacts, combined with power loss as the heat source, to calculate the temperature distributions of the belt and pulleys. Other studies have focused on heat analysis and thermal influence for only belts. Abe et al.^20^ developed a thermal analysis model for a typical timing belt. The analytical model considered the bending deformation of a timing belt cord as the primary source of heat generation and adopted a tire-based thermal analysis method to calculate heat generation and belt temperature. Merghache et al.^21^ proposed a thermal model for heat transfer of the AT10 timing belt. Belt–pulley heat flux was calculated using a mathematical model, and it was verified by the results of numerical simulations. Babak et al.^22^ investigated the transient heat transfer and stress wave propagation in nanocomposite sandwich plates with carbon nanotubes. Wu et al. performed experiments to investigate local and average heat transfer characteristics on the surface of the test wheel by using naphthalene sublimation technique^23^. Krane et al. developed an analytical model to calculate the temperature distribution and heat transfer capacity of a lightweight belt-type radiator for power plants^24^. This model analyzed two thermal conductions, i.e., from the drum to the belt and from the thermal dissipation of the belt to the environment, to predict temperatures under steady-state operation. Some experiments focused on the turbulence properties caused by the rotation of the solid hollow circular disc with rectangular wings insert in a circular tube^25^. Zhang et al.^26^ investigated the effects of rotating angular speed and pin configuration on the temperature maps and convective heat transfer characteristics on a rotating disk by use of numerical method. Kayhani et al. provided the analytical solutions for the heat transfer in 2-dimensional cylindrical composite laminate^27^. Merghache et al.^28^ experimentally investigated various tooth forms of synchronous belts and their impact on belt temperature. McPhee and Johnson^29^ focused on the heat transfer and dissipation for a brake disc. Belhocine and Bouchetara presented the thermal–mechanical coupled analysis of the dry contact between the brake disk and pads for the vehicle braking system^30^. While Talati and Jalalifar focused on the surface temperatures and heat partition on the pad–disk contact surface^31^. A numerical–experimental approach to calculate surface temperatures and flow partition coefficient in pad-on-disc contact application under tribological solicitations^32^, which is similar to the belt–pulley contact in this study. Detailed transient temperature calculations are presented for the thermal design of a high pressure compressor rotor of an aero-engine^33^. Song et al.^34^ developed an FEM to investigate the influence of different ambient temperatures on belt shear deformation, tension and velocity variation, and contact stress distribution during operation. Chen et al.^35^ examined the relationship between rubber belt friction and the presence or absence of an interfacial ice film under different temperatures and belt operating conditions. The heat conduction model for a two-layer cylinder and its analytical algorithm^36^ also provide useful knowledge for the thermal calculation of the pulley in this case.

However, to the best of our knowledge, the aforementioned studies have provided accurate but not efficient temperature prediction for a belt drive system. Moreover, only few works have focused on the thermal analysis of a multi-pulley belt drive system. Toward this end, this paper presents an innovative thermal model that uses an analytical algorithm to calculate thermal distribution and simplify it as a system of equations that can be solved using low computing resources. The model can provide temperature prediction within a few seconds for the optimization of part structure in the design stage; this is considerably superior to any numerical analysis method. Additionally, the above analytical algorithm considers complex pulley structures to ensure the accuracy of results. This model also can be used to inversely determine the optimal operating conditions to avoid failure and enhance the system lifecycle. Similar research also performed in various applications. Liu and Ma developed a novel 3D level-set topology optimization approach to provide the best shape and topology for parts^37^. Ning et al. adopted chip formation model and iterative gradient search method to calculate the Johnson–Cook model constants for ultra-fine-grained titanium^38^.

The remainder of this paper is organized as follows: “Research method” section describes the procedures for establishing the analytical algorithms for the thermal model to calculate the heat dissipation of pulley fins, belt–pulley interaction, and the final temperature distributions for multi-pulley systems. “Experiment” section discusses the setup and procedure of the belt drive system experiment. “Results and discussion” section presents and analyzes the experimental and analytical results to validate the proposed model. The analytical results are in good agreement with the experimental temperature data. Finally, “Concluding remarks” section concludes the paper.

## Research method

### Preliminaries

**Figure 2:**
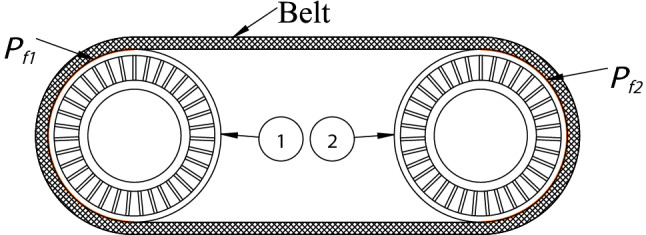
Two-pulley-one-belt system.

One preliminary for establishing a thermal model is to identify each pulley and contact surface. In an arbitrary multi-pulley belt drive system, the total number of pulleys is defined as *N*. Each pulley is assigned a value (1,2,…, *N*) in a counter-clockwise direction starting from the DR pulley in the belt layout. Further, the total number of belt–pulley contact surfaces is also *N*, and the *nth* belt–pulley contact surface denotes the contact surface between the belt and the *n*th pulley in the system. An example of a two-pulley-one-belt system is shown in Fig. 2. The left DR pulley is No. 1 and the right DN pulley is No. 2, under the condition of the left pulley driving the right pulley. The belt–pulley contact surfaces are highlighted as orange surfaces in Fig. 2.

**Figure 3:**
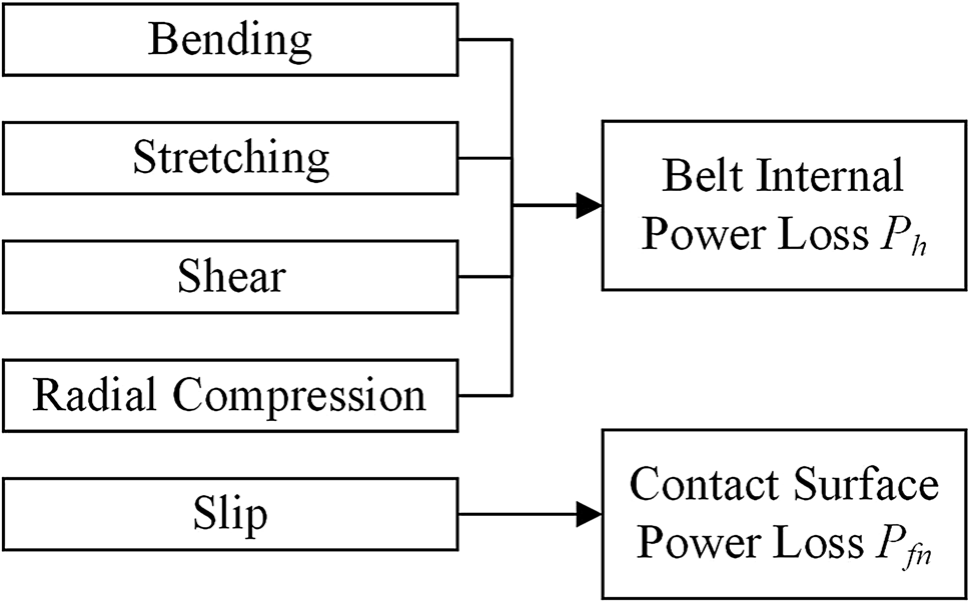
Illustration of the power loss theory.

Another preliminary for the thermal model is to calculate the heat flux generated within the belt drive system. There are five forms of energy losses^4^ according to movement mechanisms, as shown in Fig. 3. These power losses are converted into heat according to the conservation of energy. To simplify the thermal analysis of the belt drive system, these losses are subsequently classified into two types of heat sources according to their generation locations, namely, belt internal power loss *P*_*h*_^39^ inside the belt and contact surface power loss *P*_*fn*_ at the *n*th belt–pulley contact surface. Figure 2 shows that *P*_*f*1_ and *P*_*f*2_ are located at the belt–pulley contact areas and *P*_*h*_ is located inside the belt. In this study, these power losses are calculated based on the power loss algorithm developed by Gerbert, which has been used for decades.

### Overall thermal calculation

Two assumptions are made in this thermal analysis. The first is that the temperature of the belt is uniform because of the high velocity and small thickness (5–10 mm) of the belt. The second is that the thermal distribution on the pulley is tangentially homogeneous and radially symmetric owing to the high-speed spinning of the pulley and symmetric flow conditions^40–43^. The temperature gradient is along the pulley radial direction from the outer radius to the inner radius of the pulley.

**Figure 4:**
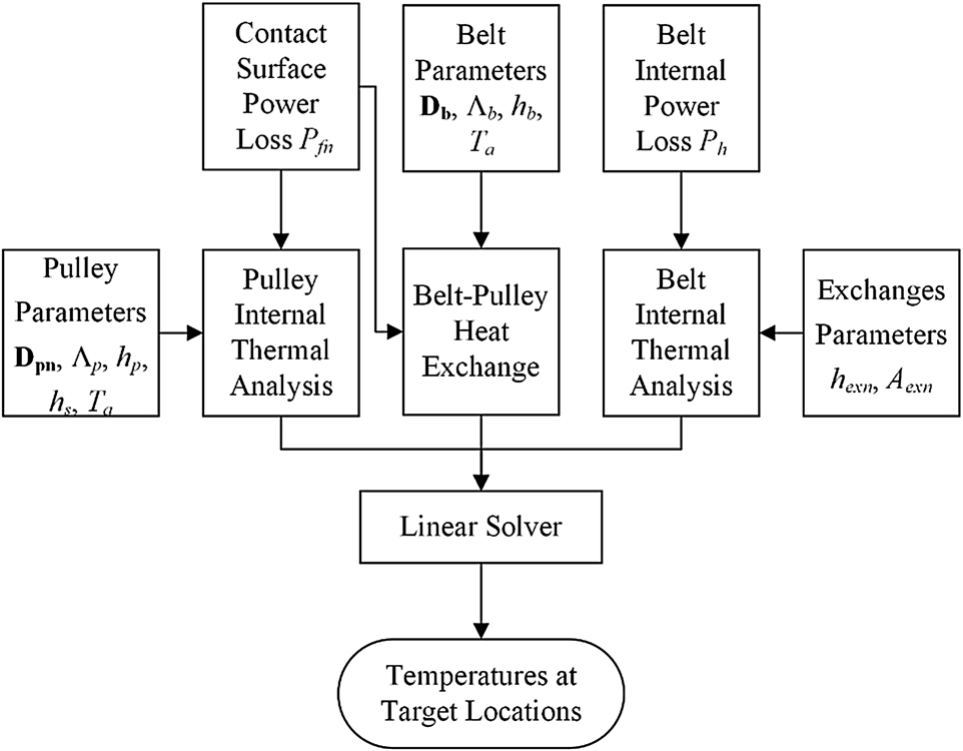
Flowchart of the method.

Figure 4 shows the general calculation procedure for the thermal model of an arbitrary multi-pulley belt drive system in this study. The first stage is to determine the heat flux and its locations within the system. Power losses *P*_*h*_ and *P*_*fn*_ are transformed into heat within the belt system. When the system runs under a constant operating condition and reaches thermal equilibrium, all generated heat is dissipated to the environment through the belt surfaces and each pulley surface.

1 $$ P_{h} + \sum\limits_{1}^{N} {P_{fn} } =\Phi _{b} + \sum\limits_{1}^{N} {\Phi _{pn} } $$

where the unknown value Φ_*b*_ is the heat dissipation flux per second from the belt surfaces to the ambient environment and the unknown value Φ_*pn*_ is the heat dissipation flux per second from the *nth* pulley surface to the ambient environment. Moreover, there is no heat source inside any pulley component (Fig. 2). A portion of *P*_*fn*_ flows into the pulley and dissipates to the environment from the pulley surfaces. Therefore, a new coefficient, *ξ*_*n*_, is introduced, which represents the percentage of *P*_*fn*_ that flows into the *nth* pulley and dissipates to the environment. Thus,

2 $$\Phi _{pn} = \xi_{n} P_{fn} $$

Combining Eqs. (1) and (2) gives

3 $$\Phi _{b} = \sum\limits_{n = 1}^{N} {\left( {1 - \xi_{n} } \right)P_{fn} } + P_{h} $$

Equation (3) implies that all generated heat that does not flow into the pulley is dissipated to the environment through the belt surfaces.

In the second stage, the model investigates the thermal behavior via three types of thermal analysis and establishes a system of equations. A function is defined for each type of analysis to illustrate the overall calculation procedure; these functions are explained later. First, function Φ_*b*_ = *f*(*T*_*b*_) is established for the belt internal thermal analysis to describe the relationship between belt surface temperature *T*_*b*_ and the heat dissipation flux per second, Φ_*b*_. This calculation is highly related to the following parameters: belt geometries matrix **D**_**b**_ (surface areas, lengths of edges and etc.), heat transfer coefficient between the belt and pulley *h*_*exn*_ and ambient temperature *T*_*a*_. The following equation is obtained by substituting this function into Eq. (3):

4 $$ \sum\limits_{n = 1}^{N} {\left( {1 - \xi_{n} } \right)P_{fn} } + P_{h} = f\left( {T_{b} } \right) $$

Second, for the belt–pulley heat exchange analysis, the temperature at the belt surface, *T*_*b*_, may be different from that at the outer radius of the *n*th pulley, *T*_*pn*_, causing pulley–belt heat flux exchange and disturbing the distribution of *P*_*fn*_ on the pulley, Φ_*pn*_. This calculation is highly related to the following parameters: contact surface area between the belt and the *nth* pulley *A*_*exn*_, thermal conductivity for the belt Λ_*b*_, heat transfer coefficient with surrounding air *h*_*b*_. The following function can be used to describe the influence of the thermal gradient (*T*_*b*_ − *T*_*pn*_) on the heat allocation of *P*_*fn*_:

5 $$\Phi _{pn} = g\left( {T_{b} - T_{pn} ,P_{fn} } \right) $$

Substituting Φ_*pn*_ with *ξ*_*n*_*P*_*fn*_ in Eq. (2) gives

6 $$ \xi_{n} P_{fn} = g\left( {T_{b} - T_{pn} ,P_{fn} } \right) $$

Third, there is a relationship between Φ_*pn*_ and *T*_*pn*_ in the pulley internal thermal analysis. This can be explained as follows: the higher the pulley surface temperature, the faster is the heat dissipation to the environment from the pulley surfaces. This calculation is highly related to the following parameters: pulley geometries **D**_**pn**_ (surface areas, lengths of edges and etc.), thermal conductivity for nth pulley Λ_*pn*_, heat transfer coefficient between the *nth* pulley and air *h*_*pn*_, and *T*_*a*_. This relationship is simply defined through a function as

7 $$\Phi _{pn} = k\left( {T_{pn} } \right) $$

Substituting Φ_*pn*_ with *ξ*_*n*_*P*_*fn*_ in Eq. (2) gives

8 $$ \xi_{n} P_{fn} = k\left( {T_{pn} } \right) $$

In the final stage, the model calculates the temperatures within the belt drive system. Equations (4), (6), and (8) form a system of equations for obtaining belt temperature *T*_*b*_ and the outer surface temperature of each pulley, *T*_*pn*_. Consider the two-pulley-one-belt system as an example. There are two pulleys and one belt in the system, and the equation set is

9 $$ \left\{ \begin{gathered} \sum\limits_{n = 1}^{2} {\left( {1 - \xi_{n} } \right)P_{fn} } + P_{h} = f\left( {T_{b} } \right) \\ \xi_{1} P_{f1} = g\left( {T_{b} - T_{p1} ,P_{f1} } \right) \\ \xi_{2} P_{f2} = g\left( {T_{b} - T_{p2} ,P_{f2} } \right) \\ \xi_{1} P_{f1} = k\left( {T_{p1} } \right) \\ \xi_{2} P_{f2} = k\left( {T_{p2} } \right) \\ \end{gathered} \right. $$

There are five unknown values in these five equations, namely, *T*_*p*1_, *T*_*p*2_, *T*_*b*_, *ξ*_1_, and *ξ*_2_; hence, there is only one solution. When a third pulley is added to this system, there will be additional versions of Eqs. (6) and (8) in Eq. (9), which ensures that the two additional unknown values, *T*_*p*3_ and *ξ*_3_, have only one solution. Extra pulleys can be added to the system in the same manner. By utilizing this technique, this model can calculate the temperature for an arbitrary belt drive system.

However, functions *f*(*T*_*b*_)*, g*(*T*_*b*_ − *T*_*pn*_*, T*_*pn*_)*,* and *k*(*T*_*pn*_) must be established to make this algorithm functional. The details of establishing these functions are presented below.

#### Belt inner calculation

The belt dissipates heat to the ambient environment during its operation. There is one assumption that the temperature inside the belt is assumed to be uniform because it has a simple structure with a high length-to-thickness ratio. Consequently, this study uses *T*_*b*_ to represent the surface and inner temperature of the belt. Consequently, the relationship between the heat dissipation rate from the belt surfaces, Φ_*b*_, and belt temperature *T*_*b*_ is given by

10 $$\Phi _{b} = f\left( {T_{b} } \right) = A_{b} h_{b} \left( {T_{b} - T_{a} } \right) $$

where *A*_*b*_ is the total belt surface area exposed to the environment, *h*_*b*_ is the heat transfer coefficient of the belt rubber, and *T*_*a*_ is ambient temperature. Combining Eq. (10) with Eq. (3) gives

11 $$ \sum\limits_{n = 1}^{N} {\left( {1 - \xi_{n} } \right)P_{fn} } + P_{h} = A_{b} h_{b} \left( {T_{b} - T_{a} } \right) $$

#### Individual pulley–belt heat exchange calculation

The heat analysis at the belt–pulley engaged surfaces is complex. The *nth* belt–pulley engaged surface involves not only heat flux exchanges between the belt and pulley but also the distribution of *P*_*fn*_ to the belt and pulley. The heat exchange flux per second at the *nth* belt–pulley engaged surface (Φ_*exn*_) from the belt to the *nth* pulley is related to the pulley–belt temperature difference (*T*_*b*_ − *T*_*pn*_) as follows:

12 $$\Phi _{exn} = A_{exn} h_{exn} \left( {T_{b} - T_{pn} } \right) $$

where *A*_*exn*_ is the area of the *nth* belt–pulley engaged surface and *h*_*exn*_ is the thermal contact conductance coefficient.

Moreover, Φ_*exn*_ influences the heat allocation of *P*_*fn*_ at the belt–pulley contact surfaces. When *T*_*pn*_ = *T*_*b*_, the pulley acquires half of the frictional heat and *ξ*_*n*_ = 0.5. There is no heat exchange between the belt and pulley in this situation. When *T*_*pn*_ < *T*_*b*_, heat flows from the belt to the pulley and the pulley acquires additional Φ_*exn*_, making *ξ*_*n*_ > 0.5. Hence, this phenomenon can be expressed as

13 $$\Phi _{exn} = \left( {\xi_{n} - 0.5} \right)P_{fn} $$

Combining Eqs. (12) and (13) yields the following equation, which provides the details of function *g*(*T*_*b*_ − *T*_*pn*_*, P*_*fn*_) in Eq. (5).

14 $$ \xi_{n} P_{fn} = A_{exn} h_{exn} \left( {T_{b} - T_{pn} } \right) + 0.5P_{fn} $$

#### Individual Pulley inner thermal calculation

**Figure 5:**
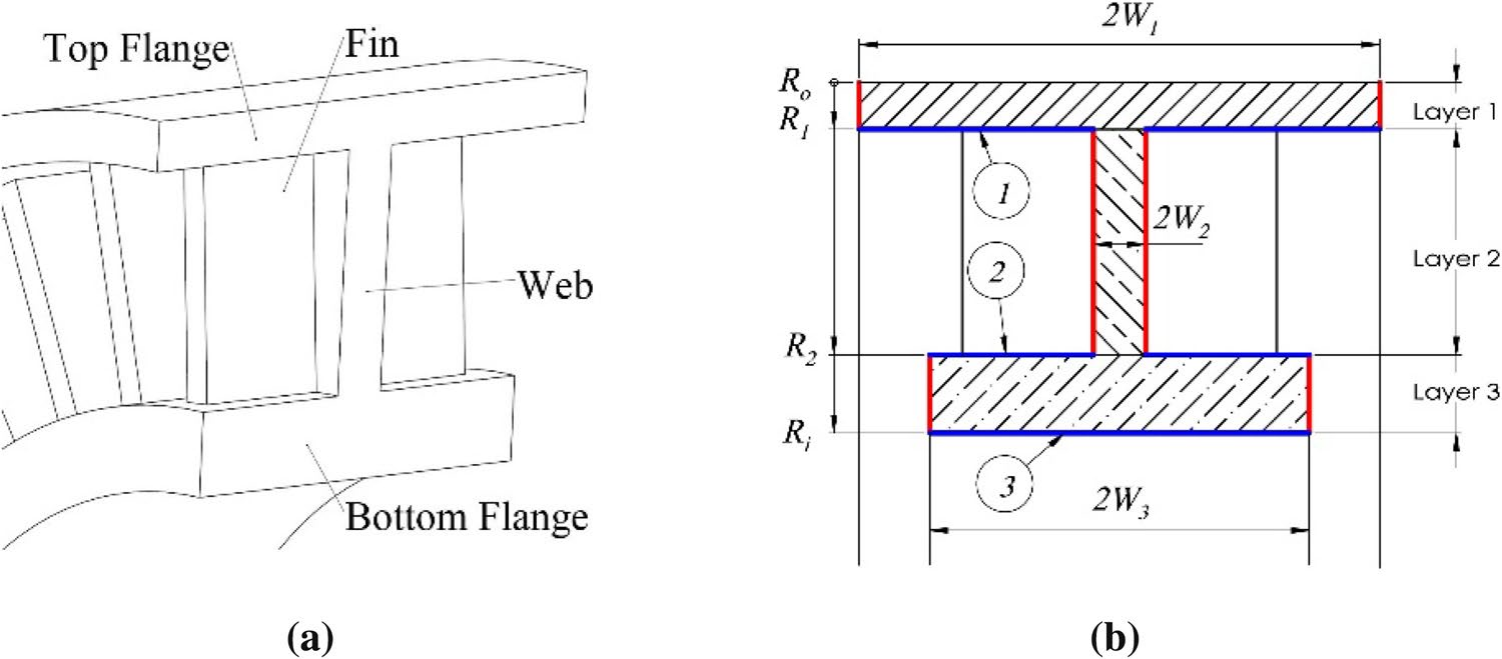
Structure of a general pulley.

A general industrial pulley can be simplified by suppressing the irrelevant chamfer and fillet features. Its cross section is shown in Fig. 5a. It consists of top and bottom flanges and a middle web section attached by fins on two sides to improve structural stiffness under high belt hub load.

One assumption is made in the thermal calculation for the pulley structure. Owing to the pulley rotates at high-speed spinning during the operation, the temperature profiles are tangentially homogeneous and radially symmetrical distribution^40,42,43^. Therefore, the assumption is that the temperatures on two flanges and the middle web are uniform in the axial direction of a pulley. On the other hand, this assumption does not apply to the temperature profile on the fins because of their dramatic surface heat dissipations and large temperature decrease in the pulley’s axial direction. This assumption is only applicable for the well-structured cross-sections such as I, L or C shapes. However, these cross-sections account for 95% of the current industrial pulleys used in engine belt drive, making this model widely used in the industrial world.

The pulley can be divided into three layers, as shown in Fig. 5b. Layer 1 is the top flange, layer 2 is the web with two fin sections, and layer 3 is the bottom flange. In this structure, a belt and a shaft with a uniform temperature on their surfaces are in contact with the top and bottom flanges of the pulley, making the temperatures on both flanges uniform along the pulley axial direction as well. Moreover, the web in layer 2 is extremely thin; hence, the temperature gradient in the pulley axial direction is neglected. As a result, the temperature in the hatched areas is considered uniform along the pulley axial direction.

**Figure 6:**
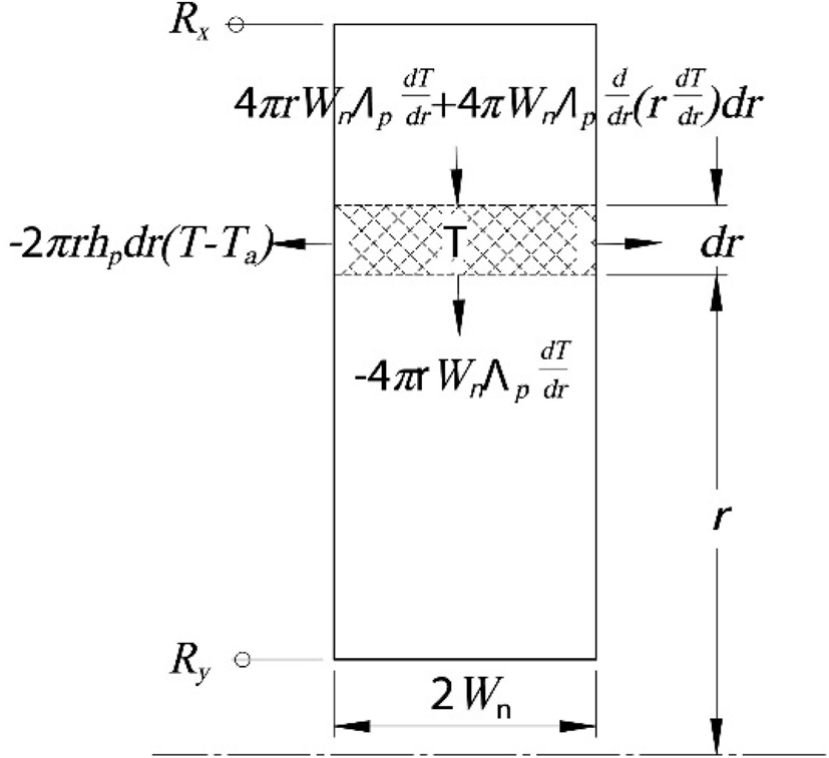
Thermal behavior at one cross section.

All three layers have rectangular cross sections highlighted as three different hatched areas in Fig. 5b. Consider one rectangular cross section in the *i*th layer as an example. The outer and inner radii are defined as *R*_*x*_ and *R*_*y*_, respectively. Figure 6 shows the thermal behavior in this Sect. ^44^.

The balance of heat gives

15 $$ 4\pi W_{i}\Lambda _{p} \frac{d}{dr}\left( {r\frac{dT}{{dr}}} \right)dr = 2 \cdot 2\pi rh_{p} dr\left( {T - T_{a} } \right) $$

Function *T*(*r*) describes the temperature distribution along the pulley radial direction, *W*_*i*_ is the half width of the *i*th layer, Λ_*p*_ is the thermal conductivity of the pulley material, and *h*_*p*_ is the heat transfer coefficient between the pulley surface and ambient environment. Equation (15) changes to an ODE as follows:

16 $$ \frac{{d^{2} T}}{{dr^{2} }} + \frac{1}{r}\frac{dT}{{dr}} - \frac{{h_{p} }}{{W_{i}\Lambda _{p} }}\left( {T - T_{a} } \right) = 0 $$

**Figure 7:**
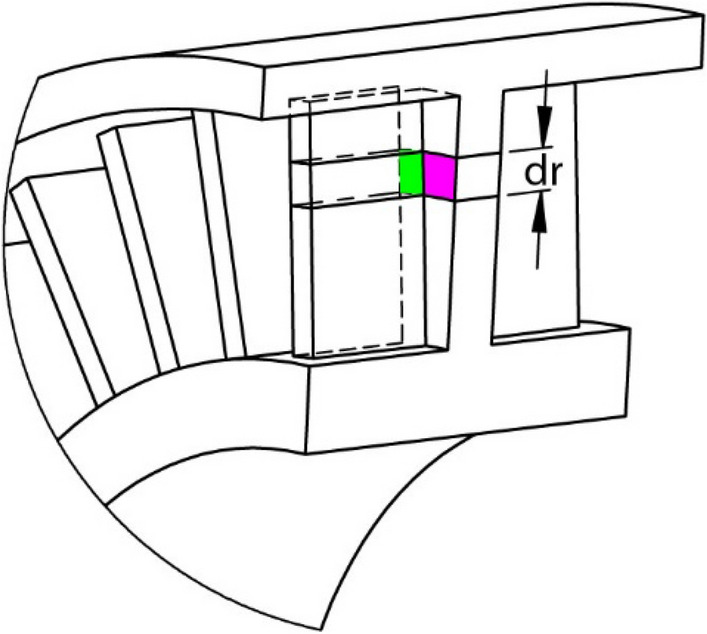
Two areas with and without connection to a fin.

However, in this study, as layer 2 consists of fins on both sides, Eq. (16) has a limitation and the thermal model must consider the heat dissipation on the fin surfaces. For a differential increase, *dr*, shown in Fig. 7, a certain amount of heat flows into the fin through the web–fin connecting area (green area in Fig. 7) and then dissipates to the environment through the fin surfaces. This area has an amplified heat dissipation rate owing to the fin, while the remaining areas (purple area in Fig. 3) have the natural heat dissipation rate, *h*_*p*_, of the pulley material. Therefore, this study introduces a new coefficient, *η*_*f*_, to indicate that the heat dissipation rate in the dashed area increases to *η*_*f*_·*h*_*p*_. When the number of fins connected to the web on one side is *N*_*f*_ in the cylinder body of differential *dr*, the dissipation rate on one side of the body is [*N*_*f*_⋅*W*_*f*_⋅*dr*⋅*η*_*f*_⋅*h*_*p*_ + (2π*r*⋅*dr* − *N*_*f*_·*W*_*f*_·*dr*)·*h*_*p*_]. However, the original dissipation rate is *2πr*⋅*dr*⋅*h*_*p*_ if there is no fin attached. Therefore, *h*_*p*_ on the web side surfaces is amplified by a factor of [1 + *N*_*f*_⋅*W*_*f*_⋅(*η*_*f*_ − 1)/2π*r*]. Equation (16) is modified to

17 $$ \frac{{d^{2} T}}{{dr^{2} }} + \frac{1}{r}\frac{dT}{{dr}} - \frac{{\left[ {1 + \frac{{N_{f} W_{f} \left( {\eta_{f} - 1} \right)}}{2\pi r}} \right]h_{p} }}{{W_{i}\Lambda _{p} }}\left( {T - T_{a} } \right) = 0 $$

The equation is applicable to the layers with and without fins. When there is no attached fin, such as in layers 1 or 3, *η*_*f*_ is equal to 1 and Eq. (15) reverts to Eq. (14). When fins are attached, such as in layer 2, *η*_*f*_ > 1 and Eq. (15) considers the extra heat dissipation on the fin surfaces. To solve Eq. (15), the key factor is to determine the value of *η*_*f*_ and whether *η*_*f*_ varies along pulley radius *r*.

**Figure 8:**
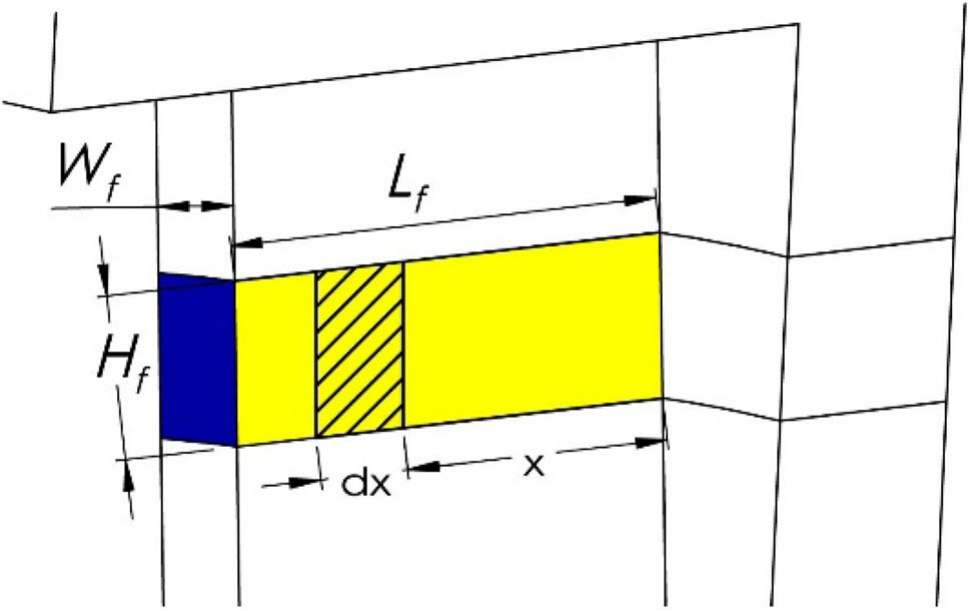
Geometry of the fin.

The fin connected to the cylinder body of differential *dr* can be considered as shown in Fig. 8. The temperature on the plate decreases from the web end to the edge end because of heat dissipation. The general heat dissipation equation for this flat plate is^45^

18 $$ \frac{{\partial^{2} G}}{{\partial x^{2} }} = - \frac{{2h_{p} H_{f} \left( {G - T_{a} } \right)}}{{\Lambda _{p} A_{fc} }} $$

where *A*_*fc*_ is the connected area (green area in Fig. 7) between the fin and web, *G*(*0*) is the temperature at *A*_*fc*_, which is equal to *T*(*r*), and *G*(*x*) is the temperature distribution function along the pulley axial direction. The analytical solution of Eq. (18)^45^ provides the temperature distribution from 0 to fin length *L*_*f*_:

19 $$ \left\{ \begin{gathered} G\left( x \right) = \frac{{\left[ {G\left( 0 \right) - T_{a} } \right]\cosh \left[ {m \cdot \left( {L_{f} - x} \right)} \right]}}{{\cosh \left( {m \cdot L_{f} } \right)}} + T_{a} \hfill \\ m = \sqrt {\frac{{2h_{p} H_{fin} }}{{\Lambda _{p} A_{fc} }}} \hfill \\ \end{gathered} \right. $$

The total heat dissipation for each fin is the summation of the heat dissipation flux per second on the fin end surfaces, Φ_*fs*_ (dark blue surface in Fig. 8), and the heat dissipation flux per second on the two side surfaces of the fin, Φ_*fe*_ (yellow surface in Fig. 8):

20 $$\Phi _{fin} =\Phi _{fs} +\Phi _{fe} = \mathop \smallint \limits_{0}^{{L_{f} }} 2 \cdot H_{f} \cdot h_{p} \cdot G\left( x \right)dx + h_{p} \cdot A_{fc} \cdot \left[ {G\left( {L_{f} } \right) - T_{a} } \right] $$

Therefore, the fin coefficient can be calculated according to its definition:

21 $$ \eta_{f} = \frac{{\Phi _{fin} }}{{\Phi _{Afc} }} = \frac{{\Phi _{fin} }}{{h_{p} \cdot A_{fc} \cdot \left[ {G\left( 0 \right) - T_{a} } \right]}} $$

The above-mentioned calculation implies that *η*_*f*_ does not change under various web surface temperatures, *G*(*0*), when the system is in thermal equilibrium; hence, it is not influenced by radius *r* or *T*(*r*). The fin dimensions (*W*_*f*_, *L*_*f*_) and heat dissipation flux per second (*h*_*p*_) are the only parameters required for calculating *η*_*f*_. Thus, for each pulley, *η*_*f*_ is a constant in Eq. (17).

As *η*_*f*_ is independent of *r*, Eq. (17) can be regarded as Kummer’s equation^46^. Its analytical solution, *T*(*r*), provides the temperature distribution of the *i*th layer as follows:

22 $$ \left\{ \begin{gathered} T\left( r \right) = F_{1} \left( r \right) \cdot C_{1} + F_{2} \left( r \right) \cdot C_{2} + T_{a} \hfill \\ \hfill \\ F_{1} \left( r \right) = {\text{e}}^{{ - \frac{{\sqrt {h_{p} } r}}{{\sqrt {W_{i}\Lambda _{p} } }}}} KummerM\left[ {\frac{{\frac{{\sqrt {h_{p} } N_{f} W_{f} \left( {\eta_{f} - 1} \right)}}{2} + \pi \sqrt {\Lambda _{p} W_{i} } }}{{2\pi \sqrt {\Lambda _{p} W_{i} } }},1,\frac{{2\sqrt {h_{p} } r}}{{\sqrt {W_{i}\Lambda _{p} } }}} \right] \hfill \\ F_{2} \left( r \right) = {\text{e}}^{{ - \frac{{\sqrt {h_{p} } r}}{{\sqrt {W_{i}\Lambda _{p} } }}}} KummerU\left[ {\frac{{\frac{{\sqrt {h_{p} } N_{f} W_{f} \left( {\eta_{f} - 1} \right)}}{2} + \pi \sqrt {\Lambda _{p} W_{i} } }}{{2\pi \sqrt {\Lambda _{p} W_{i} } }},1,\frac{{2\sqrt {h_{p} } r}}{{\sqrt {W_{i}\Lambda _{p} } }}} \right] \hfill \\ \end{gathered} \right. $$

where constant coefficients *C*_1_ and *C*_2_ can be derived from the thermal analysis boundary conditions. The temperature at the inner diameter, *T*(*R*_*y*_), is not infinity and then *C*_2_ = 0. Therefore, *C*_1_ can be calculated as

23 $$ \left\{ \begin{gathered} r = R_{x} \Rightarrow C_{1} = \frac{{T\left( {R_{x} } \right) - T_{a} }}{{F_{1} \left( {R_{x} } \right)}} \hfill \\ r = R_{y} \Rightarrow T\left( {R_{y} } \right) = {\text{limited}};C_{2} = 0 \hfill \\ \end{gathered} \right. $$

The temperature distribution for one layer can be obtained as

24 $$ T\left( r \right) = \frac{{F_{1} \left( r \right)}}{{F_{1} \left( {R_{x} } \right)}}\left[ {T\left( {R_{x} } \right) - T_{a} } \right] + T_{a} $$

Assume that *T*_*pn*_ or the temperature at the outer radius of layer 1 is known. The temperature distribution of a pulley can be calculated using Eq. (24) three times from layer 1 to layer 3. The connection of two layers shares the same temperature, and hence, *T*(*R*_*y*_) in one layer is equal to *T*(*R*_*x*_) in the next layer.

Subsequently, the heat dissipation rate at the two side surfaces in the *i*th layer is related to the integration of surface temperature distribution.

25 $$ \left\{ \begin{aligned} K\left( {r,i} \right) & = 2\int {h_{p} } \left[ {2\pi r + N_{f} W_{f} \left( {\eta_{f} - 1} \right)} \right]\left[ {T\left( r \right) - T_{a} } \right]{\text{d}}r \\ & = F_{1} \left( r \right)\sqrt {\frac{{N_{f} W_{f} }}{{h_{p} }}} \left\{ {\left( {T_{p} - T_{a} } \right)\left\{ {h_{p} N_{f} W_{f} \left[ {F_{1} \left( r \right) - F_{3} \left( r \right)} \right]\left( {\eta_{f} - 1} \right)} \right.} \right. \\ & \quad \left. {\left. { + 2\pi h_{p} rF_{1} \left( r \right) + 2\sqrt {h_{p}\Lambda _{p} W_{i} } \left[ {F_{1} \left( r \right) - \pi F_{3} \left( r \right)} \right]} \right\}} \right\} \\ F_{3} \left( r \right) & = {\text{e}}^{{ - \frac{{\sqrt {h_{p} } r}}{{\sqrt {W_{i}\Lambda _{p} } }}}} KummerM\left[ {\frac{{\frac{{\sqrt {h_{p} } N_{f} W_{f} \left( {\eta_{f} - 1} \right)}}{2} + \pi \sqrt {\Lambda _{p} W_{i} } }}{{2\pi \sqrt {\Lambda _{p} W_{i} } }} + 1,1,\frac{{2\sqrt {h_{p} } r}}{{\sqrt {W_{i}\Lambda _{p} } }}} \right] \\ \end{aligned} \right. $$

In function *K*(*r,i*), input parameter *i* determines the value of *W*_*i*_. The total heat dissipated per second at the side surfaces of all three layers (red surfaces in Fig. 5b), Φ_*ps*_, is calculated layer by layer. It is given by

26 $$\Phi _{ps} = \left. {K\left( {r,1} \right)} \right|_{{R_{o} }}^{{R_{1} }} + \left. {K\left( {r,2} \right)} \right|_{{R_{1} }}^{{R_{2} }} + \left. {K\left( {r,3} \right)} \right|_{{R_{2} }}^{{R_{i} }} $$

where *R*_*o*_ and *R*_*i*_ are the outer and inner radii of the investigated pulley, respectively, and *R*_1_ and *R*_2_ are the inner radii of layers 1 and 2, respectively, as shown in Fig. 5b. Furthermore, this model includes the heat dissipated from other surfaces, Φ_*pb*_ (blue surfaces marked as 1, 2, and 3 in Fig. 5b), which have a uniform temperature:

27 $$\Phi _{pb} = A_{1} h_{p} \left[ {T\left( {R_{1} } \right) - T_{a} } \right] + A_{2} h_{p} \left[ {T\left( {R_{2} } \right) - T_{a} } \right] + A_{3} h_{s} \left[ {T\left( {R_{i} } \right) - T_{a} } \right] $$

where *A*_1_, *A*_2_, and *A*_3_ are the surfaces areas identified by balloons 1, 2, and 3 in Fig. 5b, and *h*_*s*_ is the heat transfer coefficient between the pulley surface and shaft material. Therefore, the total heat dissipation rate of the investigated pulley is

28 $$\Phi _{pn} =\Phi _{pb} +\Phi _{ps} $$

It is impossible to calculate Φ_*pn*_ without the value of *T*_*pn*_. However, based on the above equations, Φ_*pn*_ is directly proportional to *T*_*pn*_ when the belt drive system is under thermal equilibrium. A new coefficient, ***λ***_*pn*_, is introduced for the *nth* pulley to connect these two values.

29 $$ \lambda_{pn} = \frac{{\Phi _{pn} }}{{T_{pn} }} $$

Substituting Φ_*pn*_ with *ξ*_*n*_*P*_*fn*_ in Eq. (2) and function *k*(*T*_*pn*_) in Eq. (8) gives

30 $$ \xi_{n} P_{fn} = \lambda_{pn} \cdot T_{pn} $$

#### Global system thermal calculation

Combining Eq. (9) with the functions in Eqs. (11), (14), and (30) provides the following mathematical model for an arbitrary multi-pulley belt drive system:

31 $$ \left\{ \begin{gathered} \sum\limits_{n = 1}^{N} {\left( {1 - \xi_{n} } \right)P_{fn} } + P_{h} = A_{b} h_{b} \left( {T_{b} - T_{a} } \right) \\ \xi_{1} P_{f1} = A_{ex1} h_{ex1} \left( {T_{b} - T_{p1} } \right) + 0.5P_{f1} \\ \vdots \\ \xi_{N} P_{fN} = A_{exN} h_{exN} \left( {T_{b} - T_{pN} } \right) + 0.5P_{fN} \\ \xi_{1} P_{f1} = \lambda_{p1} \cdot T_{p1} \\ \vdots \\ \xi_{N} P_{fN} = \lambda_{pN} \cdot T_{pN} \\ \end{gathered} \right. $$

The belt drive system in an engine generally contains *N* pulleys depending on the design of the engine and its parts. Equation (31) is a system of equations with a maximum of 2(*N* + 1) unknown variables (*T*_*p*1_,…, *T*_*pN*_, *ξ*_1_,…,*ξ*_*N*_ and *T*_*b*_). This study adopts LU decomposition^47^ to solve Eq. (31). In addition, the temperature distribution of a pulley in this system can be calculated layer by layer according to Eq. (24) when the value of *T*_*pn*_ is known.

Even though this model involves numerous ODEs and integrations, the analytical solution of each ODE is adopted. Moreover, the model is reduced to a system of equations in the final stage. Only a few seconds are required to obtain the predicted temperature distribution on a typical computer. Therefore, this model can predict the results efficiently.

## Experiment

The purpose of this experiment is to validate the accuracy of the proposed thermal model by measuring the thermal distribution of the entire belt drive and comparing it with the results obtained by the thermal model under various operating conditions. A rigorous experimental arrangement is necessary before validating the thermal algorithm with the multi-pulley configuration at an elevated temperature.

### Equipment and layout setup

**Figure 9:**
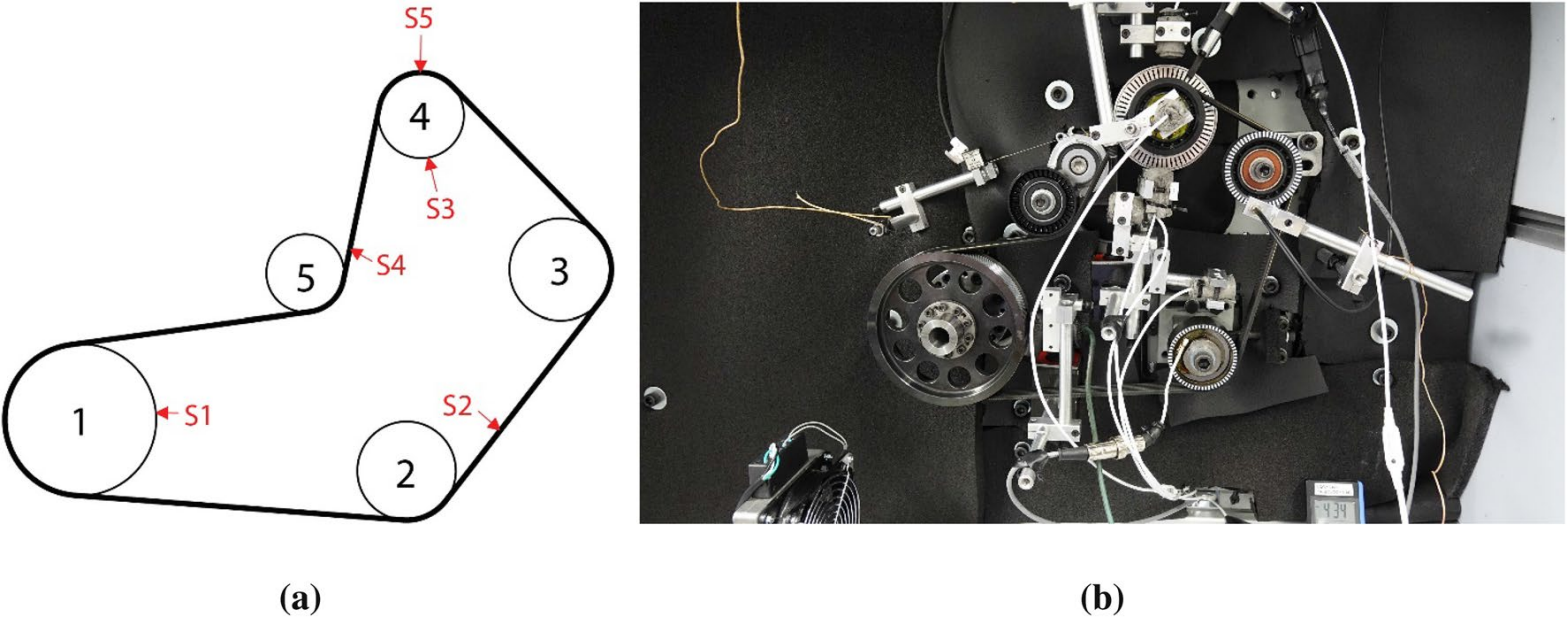
Belt layout and its installation in the experimental belt system.

First, a five-pulley belt drive system is designed (Fig. 9a) and installed on an engine simulator (Fig. 9b)^48^. Table 1 summarizes the information of this belt drive layout. In this layout, pulley1 is used as the DR pulley, pulley4 is used as the DN pulley, pulley2 contains a hub load sensor under it to measure belt tension, pulley3 is used to measure belt speed, and pulley5 is a tensioner pulley used to reduce the vibration of the belt and keep belt tension constant. In addition, thermal measurement devices are installed close to the measurement locations. Five infrared sensors measure the temperatures at the selected locations, marked S1 to S5 (Fig. 9a). These sensors are the Micro-Epsilon CSmi-SF15-C1^49^. The measurement range of these sensors is from − 40 to 1030 °C. Its accuracy is within ± 1.5%. A thermal graphic camera is installed to target the DN pulley and measure its temperature distribution during the test. It is FLIR E60. The measurement range of this camera is from − 20 to 130 °C, and its accuracy is within ± 2%. Both types of measure devices meet the requirements of this study because the maximum temperature in this experiment is 121 °C. Further, an ancillary data acquisition system records the values measured by the sensors.

**Table 1:** Layout of tested belt drive system.

Pulley ID	X (mm)	Y (mm)	Radius (mm)
1	0	0	148
2	257	− 20	65
3	290	178	62
4	193	209	62
5	95	122	65

### Load case

This experiment also considers the influence of operating conditions on temperature. A wide range of test operating conditions of the belt drive system is vital for validating the results of the thermal model in all aspects. Engine operation varies significantly from idle to the maximum revolution/torque conditions, and it is represented through three parameters: transmitted speed, transmitted torque, and ambient temperature. Table 2 summarizes the selected test points to fully consider the significant variation in engine operating conditions. Only one parameter is changed in each test, and hence, the total number of tests is 12. To investigate the influence of ambient temperature on the temperature distribution of the belt drive system, the ambient temperature in the warm start (WS) engine condition is twice as high as that in the cold start (CS) engine condition. The experiment controls the transmitted speed and torque of the belt drive system by altering the revolution and torque load of the DR pulley. In addition, a customized insulated chamber mounted on the engine dynamometer encloses the belt drive system, and engine ambient temperature is simulated by adjusting internal chamber temperature. Two thermocouples mounted at the two sides of the chamber monitor internal temperature, and a case fan inside the chamber ensures that chamber temperature is uniformly distributed. The temperature distributions of the belt system are measured under the designed working conditions for validating the thermal model using the above devices and equipment as a test bench.

**Table 2:** Test operating parameters.

Parameter	Symbol	Variation
Transmitted speed (RPM)	*ω*_*s*_	600/900/1200
Transmitted torque (*N* *m*)	*τ*_*s*_	12/17
Ambient temperature	*T*_*a*_	Cold start (CS)/warm start (WS)

### Conduction of experiment

The operation of this experiment is to perform the temperature measurements under the different test operating conditions. There is 12 configurations base on Table 2. There are three tests performed in each configuration to reduce the error. In each test, the system requires about 10–20 min to reach the thermal equilibrium status regarding the various torque and speed loads. The temperature data are recorded three times after the readings from sensors are stable. The measured values are averaged before the comparison and validation for the thermal model.

At the same time, the thermal model uses the above-mentioned information related to the belt layout and operating conditions, along with the pulley geometries and thermal properties (*h*_*p*_, Λ_*p*_, …) measured using standard procedures, to calculate the temperature distributions of the belt drive system under the designed operating conditions. The comparison between experimental and analytical results is presented in the following section.

## Results and discussion

### Assumption verification

The two assumptions used in the development of the thermal model are the fundamentals of this model. Hence, it is crucial to verify these two assumptions through experiments to ensure the feasibility of the method employed by this model.

**Figure 10:**
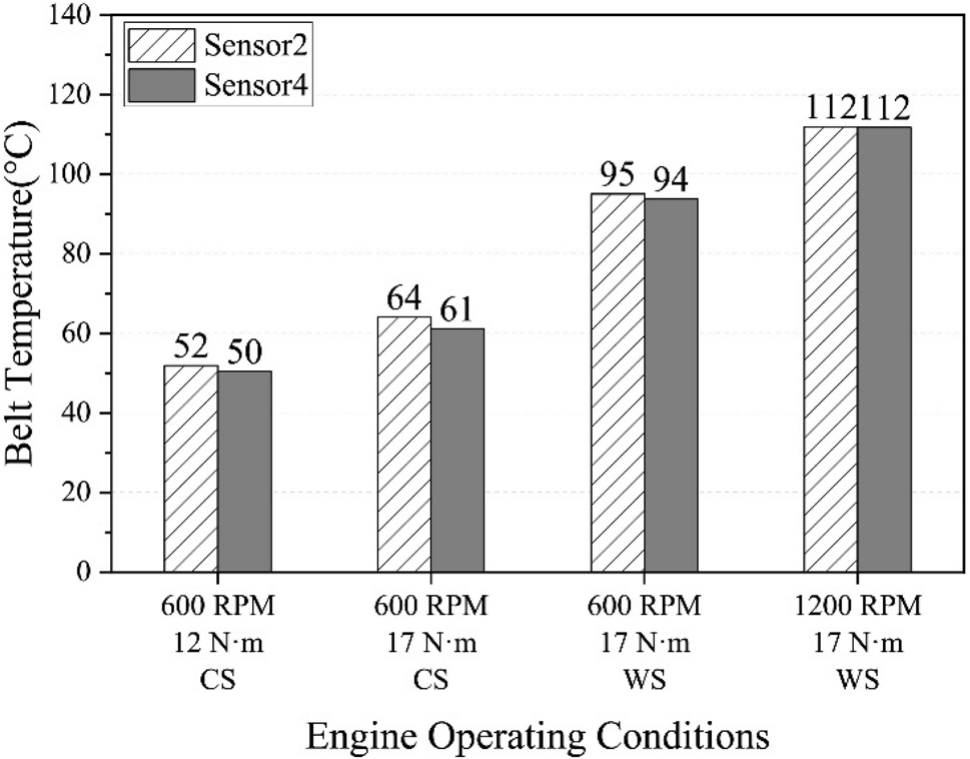
Temperatures at two locations on belt surfaces under various engine operating conditions.

The assumed uniform belt temperature in the thermal model can be verified by the temperatures measured at two different locations on the belt: sensor 2 and sensor 4 (Fig. 9a). Figure 10 shows the temperatures measured at these two locations under four different belt drive conditions. The differences in these measurements are less than 3 °C, indicating that it is feasible to assume that the belt has a uniform temperature during high-speed movement. Hence, these measured temperatures are considered as belt temperatures under different working conditions.

**Figure 11:**
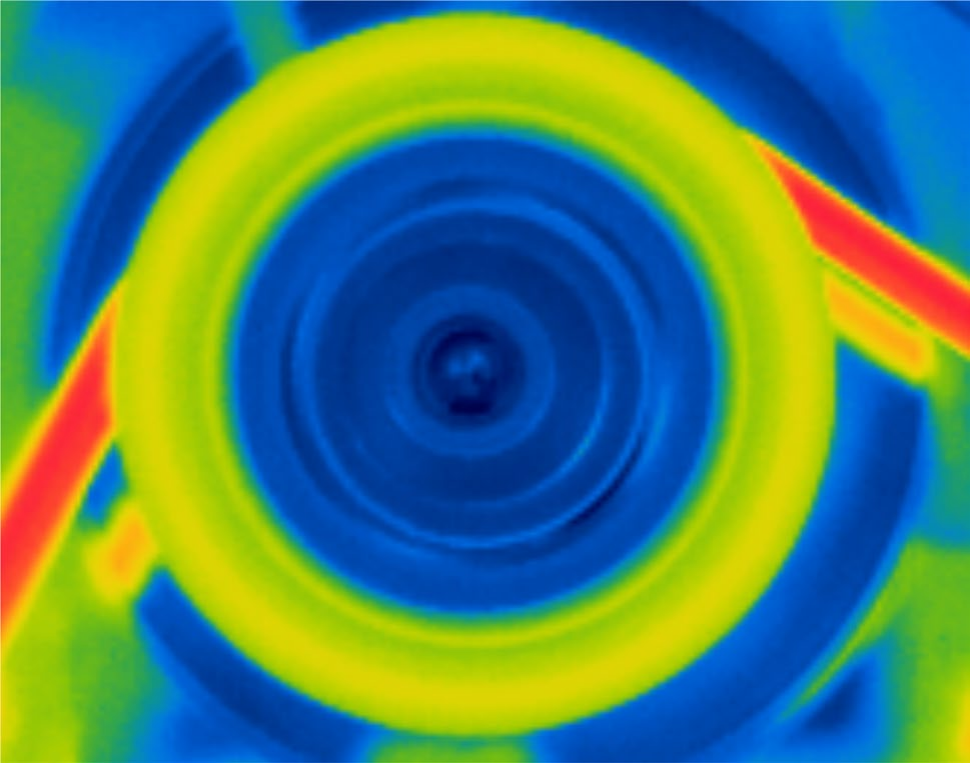
Tangentially homogeneous and radially symmetric temperature distribution of a high-speed FRP pulley.

Figure 11 shows the temperature distribution of a running FRP pulley with the lowest rotation speed configuration (600 RPM). A perfectly rotational symmetric temperature distribution is measured. The temperature is the highest at the outer radius, where heat is generated, and it gradually decreases in the pulley radial direction but not in the tangential direction owing to heat dissipation. Thus, the thermal distribution on the pulley is in accordance with the assumption, and the governing equations based on this assumption are feasible for this work.

### Experimental and analytical temperature verification

To validate the accuracy of the analytical results provided by the thermal model, this study compares the experimental and analytical temperatures at several selected critical locations under variations in transmitted torque and speed at the ambient conditions of CS and WS. This section describes the influence of the change in each parameter on the experimental temperature distribution and validates whether the model can predict the corresponding analytical temperature.

#### Temperature comparisons at different working loads

**Figure 12:**
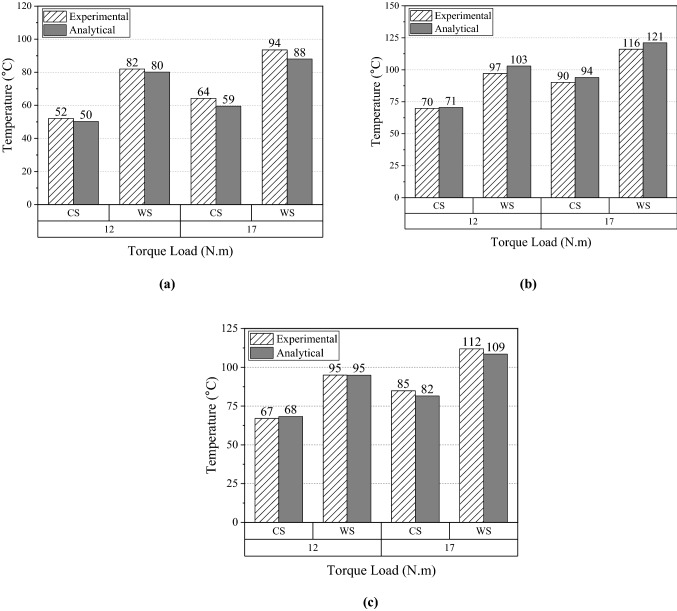
Temperature comparisons at different locations under 1200 RPM with various loads and CS and WS operating conditions. (**a**) DR pulley surface at outer radii. (**b**) DN pulley surface at outer radii. (**c**) Belt surface.

Increase in transmitted torque leads to high slip rate and heat generation, resulting in high temperatures at the belt–pulley engaged surface. The surface on the pulley side is at the outer radii of the pulleys. In this experiment, various torque loads are applied to the DR and DN pulleys. The critical positions are the belt surface and the outer radii of the DR and DN pulleys. The temperatures at these positions are plotted in detail under transmission torque loads of 12–17 N m at the ambient conditions of CS and WS, as shown in Fig. 12. The dashed column indicates the experimental temperatures, and the gray column indicates the analytical temperatures provided by the thermal model. This figure displays accurate temperature predictions by the model. The maximum difference between the experimental and analytical temperatures is 6 °C, and all differences are less than 10% of the measured temperatures.

#### Temperatures at different speeds

**Figure 13:**
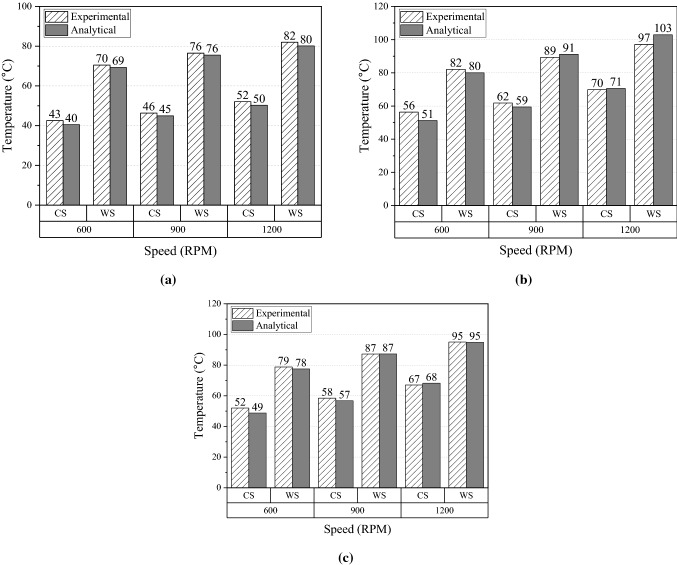
Temperature comparisons at different locations under various speeds and CS and WS ambient conditions when a transmitted torque of 12 N⋅m is applied. (**a**) DR pulley surface at outer radii. (**b**) DN pulley surface at outer radii. (**c**) Belt surface.

Another important aspect of evaluating the accuracy of the thermal model is to compare the analytical and experimental temperatures under different transmitted speeds. Unlike the effect of torque change, variation in speed alters not only heat generation but also the heat dissipation on exposed surfaces in the belt drive system. Figure 13a–c shows the analytical temperatures at the outer radius of the DR and DN pulleys and the belt surface under the ambient conditions CS and WS with a fixed transmission load of 12 N m and speed ranging from 600 to 1200 RPM. The temperatures on the pulley outer surfaces and belt surface increase with speed. This indicates that the increase in heat generation is more significant than that in the heat transfer coefficients. The small differences between the experimental and analytical results shown in Fig. 13 demonstrate that the thermal model can accurately capture this trend of temperature increase. The maximum temperature difference is only 5 °C, or 10% of the measured results.

#### Temperatures distributions of the pulley

**Figure 14:**
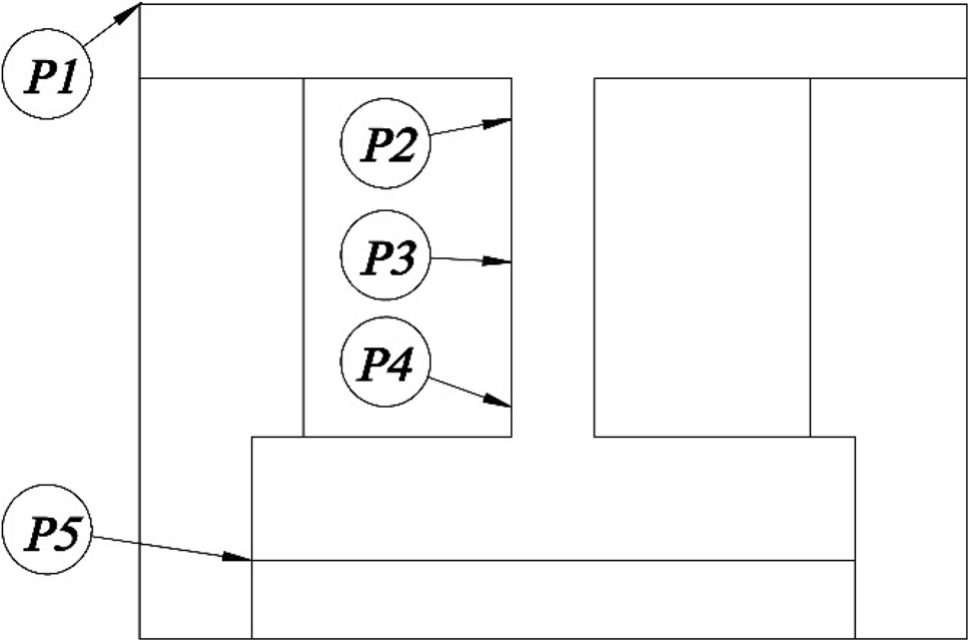
Selected points along the pulley radius.

**Figure 15:**
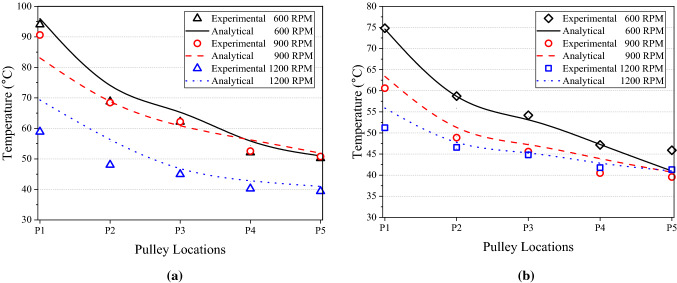
Comparison of experimental and analytical temperature distributions at P1–P5 under the CS condition for (**a**) DR pulley and (**b**) DN pulley.

The temperature distribution of a pulley is important because it is highly related to the heat dissipation of the pulley which influences the accuracy of temperature predictions. The output results from the analytical model are the temperature plot along the pulley’s radial direction. On the other hand, the experimental data is recorded from the five selected points at different radii on the pulley surfaces, as shown in Fig. 14. As for resultant comparisons, the predicted and experimental temperature distribution of the DR and DN pulleys under various speed conditions is displayed in Fig. 15. Based on these two comparisons, the model successfully predicts the decrease in temperature from the outer diameter to the inner diameter in all circumstances, and the predicted temperatures are similar to the measured values. With an accurate temperature plot for the pulley, the thermal model can calculate the heat dissipation and λpn for each pulley. Then, the system of equations can accurately predict the temperatures at pulley surfaces.

#### Thermal flow in the system

The thermal model provides useful information such as the thermal dissipation rate for each pulley, as shown in Table 3. The thermal dissipation rate is vital because a low thermal dissipation rate causes high heat accumulation and high temperature within a pulley, which finally leads to the thermal failure of the pulley. Hence, the thermal dissipation rate is a good indicator for a pulley in the thermal analysis. As shown in Table 3, pulleys 1 and 2 have high dissipation rates because they are fabricated from conventional steel, while the remaining three pulleys have low rates because of their FRP material, which has low thermal conductivity. Moreover, pulley 1 has a larger dissipation rate compared to pulley 2 because the diameter of pulley 1 is larger (Table 1). Furthermore, pulley 3 has a lower dissipation rate compared to pulley 2, but the values are close. This proves that pulley 3 consists of a fin section to increase heat dissipation, even though it is fabricated from FRP. In general, this table provides the impact of several parameters, including working conditions and pulley geometries and materials, on the thermal flow inside the belt drive system.

**Table 3:** Heat dissipation rate for each pulley under each CS working condition (W/s).

Conditions		Pulley ID				
		1	2	3	4	5
600 RPM	12 N m	16.36	11.83	9.09	8.32	4.45
	17 N m	15.90	15.24	12.66	10.68	4.22
900 RPM	12 N m	23.29	16.50	13.11	11.65	6.00
	17 N m	40.00	28.35	24.78	20.23	9.75
1200 RPM	12 N m	30.19	21.48	18.01	15.10	7.41
	17 N m	42.18	29.62	25.65	21.08	10.30

#### Calculation time comparison

The most important achievement of this work is that the model requires considerably less computational resources to accurately predict temperature. Table 4 indicates the times required by this developed method and other two existing methods from current literature. The multiple reference frame (MRF) and dynamic mesh models require hours and days to calculate temperature, while the proposed method only requires less than 5 s on a typical computer desktop with an Intel 4930 K CPU with 3.4 GHz and 32 GB memory. Therefore, this method is applicable for cases that consider large load, such as subsequent pulley material thermal fatigue analysis.

**Table 4:** Time required by each method to predict the temperature distribution on a belt drive.

Analytical	MRF	Dynamic mesh
5 s	16 h^18^	93 days^18^

## Concluding remarks

A novel analytical thermal model was developed to predict the temperature distributions of complex pulley geometries in a multi-pulley belt drive system in this work. This study established a model using classical ODEs for complex pulley structures to calculate the heat dissipation of pulleys. Combined with five types of heat generation within the belt drive system, this model can simulate the heat exchange among components and the thermal flows inside components. Moreover, the model was applicable for multi-pulley belt drive systems and for materials with a wide range of thermal conductivities. This work investigated a five-pulley FEAD system with steel and FRP pulleys as an example. Furthermore, the ODEs and integrations used in the model had analytical solutions; this significantly reduces computational cost and time. The time required for temperature prediction reduced from hours to seconds, thereby allowing for subsequent investigation such as thermal fatigue analysis or analyses with large load. Finally, the results provided by the model were validated by experiments performed on an engine dynamometer. The validation considered various conditions to cover a wide change of speeds and torque loads during engine operation. The predicted and experimental values generally showed good agreement in all conditions and at all points. The maximum difference between the values was less than 6 °C, which was less than 10% of the measured values. This was sufficient for ensuring the validity of the model. Future work will consider the influence of covering cases and the belt wear. In summary, this paper presented an accurate and efficient thermal model for predicting the temperature distribution of a multi-pulley belt drive system, which had considerable potential for research and industrial applications.

## Abbreviations

*A*_1_, *A*_2_, *A*_3_: Surface areas of pulley where it has a constant temperature, m^2^
*A*_*b*_: Surface area of belt exposed to the environment, m^2^
*A*_*fc*_: Connected area (green area in Fig. 7) between the fin and web, m^2^
*A*_*exn*_: Contact area between the belt and the *n*th pulley, m^2^
*C*_1_, *C*_2_: Constants for Kummer’s functions
*h*_*b*_: Heat transfer coefficient between belt and air, W/(m^2^ K)
*h*_*pn*_: Heat transfer coefficient between the *n*th pulley and air, W/(m^2^ K)
*h*_*exn*_: Thermal contact conductance coefficient at the *n*th engaged surface between the belt and the *nth* pulley in the system
*H*_*f*_: Height of the differential increase in the fin, m
*L*_*f*_: Length of the fin, m
*N*: Number of pulleys inside the belt drive system
*N*_*f*_: Number of fins of the pulley
*P*_*h*_: Heat flux generated per second in the belt in the power loss calculation, W
*P*_*fn*_: Heat flux generated per second by friction at the *n*th pulley–belt engaging surface in the power loss calculation, W
*R*_*i*_: Inner diameter of pulley, m
*R*_1_: Inner diameter of first layer, m
*R*_2_: Outer diameter of second layer, m
*R*_*o*_: Outer diameter of pulley, m
*r*: Differential increase in radius of pulley, m
*T*_*a*_: Ambient temperature of belt drive system, °C
*T*_*b*_: Uniform belt temperature, °C
*T*_*pn*_: Temperature at the outer radius of the *nth* pulley, °C
*W*_*f*_: Width of a fin, m
*W*_*i*_: Half width of the *i*th layer of the pulley, m
*x*: Differential increase along the fin, m
**D**_**b**_: Matrix storing the dimensions of the belt in the system
**D**_**pn**_: Matrix storing the dimensions of the *n*th pulley in the system
*η*_*f*_: Coefficient for fins to increase the heat transfer coefficient, $h_{p}$, for their connected areas between a fin and the web
***λ***_*pn*_: Coefficient representing the heat dissipation ability of the *nth* pulley within the system
Λ_*b*_: Thermal conductivity of belt, W/(m K)
Λ_*p*_: Thermal conductivity of pulley, W/(m K)
*ξ*_*n*_: Percentage of frictional heat flux flowing into the *n*th pulley within the system
*τ*_*s*_: Transmitted torque of the belt drive system, *N* *m*
Φ_*Afc*_: Heat dissipation flux per second through the fin connecting area without augmented dissipation on the fin, W/s
Φ_*b*_: Heat dissipation flux per second from all exposed surfaces of the belt to the environment, W/s
Φ_*exn*_: Heat exchange per second at the engaging surfaces between the belt and the *n*th pulley, W/s
Φ_*fin*_: Heat flux dissipated through the fin outer surfaces per second, W/s
Φ_*fe*_: Heat flux dissipated through the fin far-end surface per second, W/s
Φ_*fs*_: Heat flux dissipated through the fin side surfaces per second, W/s
Φ_*pn*_: Frictional heat flux flow into the pulley per second or total heat dissipation from the surfaces of a pulley or the *n*th pulley in the system per second, W/s
Φ_*pb*_: Heat dissipated per second at the surfaces on a pulley where it has a constant temperature, W/s
Φ_*ps*_: Heat dissipated per second at the side surfaces of all three layers on a pulley, W/s
*ω*_*s*_: Transmitted speed of the belt drive system, RPM

## References

[1] Kim HS, Bae HS, Yu J, Kim SY (2016). Thermal conductivity of polymer composites with the geometrical characteristics of graphene nanoplatelets. Sci. Rep..

[2] Yang Q (2018). Study of the micro-climate and bacterial distribution in the deadspace of N95 filtering face respirators. Sci. Rep..

[3] Liu X, Behdinan K (2020). Analytical-numerical model for temperature prediction of a serpentine belt drive system. Appl. Sci..

[4] Gerbert, G. *Force and slip behaviour in V-belt drives*. *Mechanical Engineering Series* **67**, (Finnish Academy of Technical Sciences, 1972).

[5] Manin, L., Liang, X. & Lorenzon, C. Power losses prediction in poly-v belt transmissions: application to front engine accessory drives. in *International Gear Conference 2014: 26th–28th August 2014, Lyon* 1162–1171 (Elsevier, 2014).

[6] Silva CAF (2017). Modeling of power losses in poly-V belt transmissions: hysteresis phenomena (standard analysis). J. Adv. Mech. Des. Syst. Manuf..

[7] Silva CAF (2018). Modeling of power losses in poly-V belt transmissions: hysteresis phenomena (enhanced analysis). Mech. Mach. Theory.

[8] Bertini L, Carmignani L, Frendo F (2014). Analytical model for the power losses in rubber V-belt continuously variable transmission (CVT). Mech. Mach. Theory.

[9] Chen TF, Lee DW, Sung CK (1998). An experimental study on transmission efficiency of a rubber V-belt CVT. Mech. Mach. Theory.

[10] Balta B, Sonmez FO, Cengiz A (2015). Speed losses in V-ribbed belt drives. Mech. Mach. Theory.

[11] Zhu H, Hu Y, Pi Y (2014). Transverse hysteretic damping characteristics of a serpentine belt: Modeling and experimental investigation. J. Sound Vib..

[12] Kim H, Marshek KM (1990). Force distribution for a flat belt drive with a concentrated contact load. Mech. Mach. Theory.

[13] Qin Z, Cui D, Yan S, Chu F (2016). Hysteresis modeling of clamp band joint with macro-slip. Mech. Syst. Signal Process..

[14] Oliver LR, Song G, Shen Y, Breig WF, Chandrashekhara K (2002). Accessory serpentine belt stress analysis using hyperelastic model. SAE Tech. Pap. Ser..

[15] Julió G, Plante JS (2011). An experimentally-validated model of rubber-belt CVT mechanics. Mech. Mach. Theory.

[16] Zhu C, Liu H, Tian J, Xiao Q, Du X (2010). Experimental investigation on the efficiency of the pulley-drive CVT. Int. J. Automot. Technol..

[17] Wurm J, Fitl M, Gumpesberger M, Väisänen E, Hochenauer C (2017). Advanced heat transfer analysis of continuously variable transmissions (CVT). Appl. Therm. Eng..

[18] Wurm J, Fitl M, Gumpesberger M, Väisänen E, Hochenauer C (2016). Novel CFD approach for the thermal analysis of a continuous variable transmission (CVT). Appl. Therm. Eng..

[19] Gerbert G (1981). Heat in V-belt Drives.

[20] Abe, S., Tokoro, M., Yaegashi, T. & Ogawa, K. Thermal analysis of timing belt. in *SAE Technical Paper Series* (1989).

[21] Merghache SM, Ghernaout MEA (2017). Experimental and numerical study of heat transfer through a synchronous belt transmission type AT10. Appl. Therm. Eng..

[22] Safaei B, Moradi-Dastjerdi R, Qin Z, Behdinan K, Chu F (2019). Determination of thermoelastic stress wave propagation in nanocomposite sandwich plates reinforced by clusters of carbon nanotubes. J. Sandw. Struct. Mater..

[23] Wu Y, Wu M, Zhang Y, Wang L (2014). Experimental study of heat and mass transfer of a rolling wheel. Heat Mass Transf..

[24] Krane RJ, Jischke MC, Rasmussen ML (1973). The thermal analysis of a belt type radiator by the method of matched asymptotic expansions. Int. J. Heat Mass Transf..

[25] Singh S, Negi JS, Bisht S, Sah H (2019). Thermal performance and frictional losses study of solid hollow circular disc with rectangular wings in circular tube. Heat Mass Transf..

[26] Zhang JZ, Tan XM, Zhu XD (2014). Investigation on convective heat transfer over a rotating disk with discrete pins. Heat Mass Transf..

[27] Kayhani MH, Shariati M, Nourozi M, Karimi Demneh M (2009). Exact solution of conductive heat transfer in cylindrical composite laminate. Heat Mass Transf..

[28] Merghache, S. M. & Ghernaout, M. E. A. Measuring the stiffness of a timing belt type binder magnetic- AT10. in *Recueil de mécanique* 71–78 (2016).

[29] McPhee AD, Johnson DA (2008). Experimental heat transfer and flow analysis of a vented brake rotor. Int. J. Therm. Sci..

[30] Belhocine A, Bouchetara M (2013). Thermal-mechanical coupled analysis of a brake disk rotor. Heat Mass Transf..

[31] Talati F, Jalalifar S (2009). Analysis of heat conduction in a disk brake system. Heat Mass Transf..

[32] Sellami A, Kchaou M, Elleuch R, Desplanques Y (2016). Thermal analysis of pad-on-disc contact under tribological solicitations: a coupled numerical–experimental approach to identify surface temperatures and flow partition coefficient. Heat Mass Transf..

[33] Hennecke DK (1984). Thermal analysis of a high-pressure compressor rotor of an aero-engine—Venting as a means for life improvement. Wärme-und Stoffübertragung.

[34] Song G, Chandrashekhara K, Breig WF, Klein DL, Oliver LR (2005). Analysis of Cord-reinforced poly-rib serpentine belt drive with thermal effect. J. Mech. Des..

[35] Chen G, Lee JH, Narravula V, Kitchin T (2012). Friction and noise of rubber belt in low temperature condition: The influence of interfacial ice film. Cold Reg. Sci. Technol..

[36] Pyr’yev, Y. Analytical solution of thermal conduction in a two-layer cylinder modeling oscillator roller in an offset machine. *Int. J. Therm. Sci.* **136**, 433–443 (2019).

[37] Liu J, Ma YS (2015). 3D level-set topology optimization: A machining feature-based approach. Struct. Multidiscip. Optim..

[38] Ning J, Nguyen V, Huang Y, Hartwig KT, Liang SY (2018). Inverse determination of Johnson–Cook model constants of ultra-fine-grained titanium based on chip formation model and iterative gradient search. Int. J. Adv. Manuf. Technol..

[39] Gerbert, B. G. *Pressure distribution and belt deformation in V-belt drives*. *J. Eng. Ind.* (1975).

[40] Pellé J, Harmand S (2009). Heat transfer study in a rotor–stator system air-gap with an axial inflow. Appl. Therm. Eng..

[41] Lallave JC, Rahman MM, Kumar A (2007). Numerical analysis of heat transfer on a rotating disk surface under confined liquid jet impingement. Int. J. Heat Fluid Flow.

[42] Polikarpova M (2015). Thermal effects of stator potting in an axial-flux permanent magnet synchronous generator. Appl. Therm. Eng..

[43] Lim DH, Kim SC (2014). Thermal performance of oil spray cooling system for in-wheel motor in electric vehicles. Appl. Therm. Eng..

[44] Gerbert.G. *Traction Belt Mechanics*. (Machine and Vehicle Design, Chalmers Univ. of Technology, 1999).

[45] Shiming Y, Wenquan T (1998). Heat Transfer.

[46] Barton DE, Abramovitz M, Stegun IA (1965). Handbook of mathematical functions with formulas, graphs and mathematical tables. J. R. Stat. Soc. Ser. A.

[47] Schwarzenberg-Czerny A (1995). On matrix factorization and efficient least squares solution. Astron. Astrophys. Suppl..

[48] Liu X, Behdinan K (2020). A novel analytical model for the thermal behavior of a fiber-reinforced plastic pulley in a front-end accessory drive. Adv. Mech. Eng..

[49] ThermoMETER Temperature Sensors Datasheet. https://www.instrumart.com/assets/CSmicro-thermoMETER-datasheet.pdf

